# A Randomized Controlled Trial of Vertebral Body Decompression Procedure Versus Conservative Treatment for Painful Vertebral Compression Fracture

**DOI:** 10.3390/medicina59101848

**Published:** 2023-10-17

**Authors:** Sanghoon Lee, Haolin Zheng, Sang-Min Park, Ho-Joong Kim, Jin S. Yeom

**Affiliations:** Spine Center and Department of Orthopaedic Surgery, Seoul National University Bundang Hospital, 82 Gumi-ro, 173 Beon-gil, Bundang-gu, Seongnam-si 13620, Republic of Korea

**Keywords:** back pain, core decompression, osteoporosis, vertebral compression fracture

## Abstract

*Background:* Traditional treatment modalities for vertebral compression fractures (VCFs) include bed rest, pain medications, muscle relaxants, back braces, and physical therapy. In cases where conservative treatment proves ineffective, a new procedure called core decompression of the vertebral body is explored. Core decompression of the vertebral body has the potential to lower and stabilize the intraosseous pressure, resulting in enhanced blood circulation, which contributes to pain reduction. In this trial, we evaluated the efficacy of core decompression of the vertebral body in patients with painful VCFs compared with conventional conservative treatment. *Methods:* This prospective randomized controlled trial was conducted at a tertiary education hospital between June 2017 and May 2020. The participants were randomly assigned in a 1:1 ratio to one of two treatment groups: the core decompression group and the conservative treatment group. The primary outcome measure was the visual analog scale (VAS) pain score of the back 3 months after the procedure. Secondary outcome measures included the Oswestry Disability Index (ODI) for lumbar disabilities, the European Quality of Life-5 Dimensions (EQ-5D) score for quality of life, and radiographic outcomes such as changes in compression rate. *Results:* All patients underwent the assigned intervention (48 core decompression and 50 conservative treatments). At both 1 month and 3 months, there were no significant differences between the core decompression group and conservative treatment group in VAS pain score (adjusted treatment effect: −0.1 and 2.0; 95% confidence interval [CI]: −7.5 to 7.3 and −5.6 to 9.6; *p* = 0.970 and *p* = 0.601, respectively). In addition, there were no significant inter-group differences in ODI and EQ-5D scores throughout the follow-up period (*p* = 0.917 and 0.704, respectively). *Conclusion:* Core decompression of the vertebral body did not demonstrate any significant improvement in pain and disability compared to conventional conservative treatment.

## 1. Introduction

Vertebral compression fractures (VCFs) can result in various complications, including chronic back pain, progressive spinal deformity, muscle wasting, pressure ulcers, and sleep disturbances [1,2,3,4]. As the population ages, osteoporotic VCFs become increasingly prevalent. Conventional treatments for VCFs typically involve bed rest, the use of oral or injectable pain medications and muscle relaxants, external back braces, and physical therapy [5,6,7,8,9]. In cases where conservative treatment is ineffective, vertebroplasty (VP) may be performed. VP is a medical procedure that involves the injection of cement into a fractured vertebra, aiming to alleviate discomfort and pain. Although it can be effective, VP is associated with several cement-related complications, including cement leakage and cement embolism. Owing to these potential side effects associated with the use of cement, we introduced a new procedure called core decompression of the vertebral body.

The underlying mechanisms responsible for pain following VCFs have not been thoroughly investigated. However, increased pressure within the bone (intraosseous pressure) is considered the primary trigger [10,11,12,13]. Based on this mechanism, we developed a core decompression procedure for vertebral bodies. Core decompression is a surgical procedure commonly used to manage osteonecrosis of the femoral head (ONFH). This procedure is typically performed by the drilling into the area of dead bone near the joint [14]. It aims to reduce pressure within the bone, thereby improving blood circulation in the affected area and providing pain relief. Similarly, core decompression of the vertebral body has the potential to lower and stabilize the intraosseous pressure for VCFs, resulting in enhanced blood circulation and pain reduction. However, there is currently no evidence regarding the effectiveness of core decompression of the vertebral body.

In this single center, randomized, controlled trial, we evaluated the efficacy of core decompression of the vertebral body in patients with painful VCFs compared with conventional conservative treatment. We hypothesized that patients who undergo core decompression of the vertebral body would report lesser pain and pain-related disabilities than those in the control group.

## 2. Materials and Methods

### 2.1. Study Design and Participants

The participants were enrolled at a tertiary education hospital between June 2017 and May 2020. This trial was approved by the institutional review board of our hospital (B-1502-288-005) and registered at ClinicalTrials.gov (NCT02902250). Before enrollment, written informed consent was obtained from each patient or their legal representatives. The trial was conducted in accordance with the principles of the Declaration of Helsinki. The clinical and radiographic data were monitored by an independent researcher.

The participants were recruited from the outpatient department of our hospital by a single specialist. The inclusion criteria were as follows: (1) aged ≥ 40 years and voluntarily agreed to participate and (2) diagnosed with one to three painful osteoporotic VCFs between vertebral levels T10 and L5. In all patients with suspected compression fractures, lumbar X-rays and MRI were performed to confirm the fracture and bone marrow edema. Also, in all patients diagnosed with VCFs, bone density scans were performed to confirm osteoporotic VCFs. The exclusion criteria were as follows: (1) previous diagnosis of an inflammatory disease or malignant tumor, (2) presence of a bleeding or coagulation disorder, (3) use of any type of anticoagulant, (4) severe pain other than in the lower back, and (5) deemed unfit for participation in this clinical trial by the researcher.

Participants who met the inclusion criteria were randomized into one of two treatment groups at a 1:1 ratio: the core decompression group and the conservative treatment group. The time from fracture to randomization was less than one month. Web-based randomization was employed to achieve concealed allocation. All participants were evaluated for the efficacy and safety of the treatment over a 3-month study period.

### 2.2. Efficacy Evaluation

At baseline, patients scored their back pain using the visual analog scale (VAS, on a scale of 0 to 100, with higher scores indicating more severe pain) and completed self-reported questionnaires, such as the Oswestry Disability Index (ODI) and the 5-level version of the European Quality of Life-5 (EQ-5D). The ODI is a widely used questionnaire designed to assess the level of disability or functional impairment experienced by individuals with low back pain. The EQ-5D score is a standardized instrument used to measure health-related quality of life. It assesses an individual’s general health and well-being and is commonly used in healthcare and clinical research to evaluate the overall health status and quality of life of patients. They also provided their demographic and clinical information. Evaluation measures were performed before randomization and at various times for up to 3 months. The primary outcome measure was the VAS pain score of the back. Secondary outcome measures included ODI and EQ-5D scores.

The compression rate and wedge angle of the vertebral body were measured on plain radiographs [15]. The heights of the anterior columns (A) of the fractured vertebral body were measured using a picture archiving and communication system (INFINITT Healthcare, Seoul, Korea). The mean of anterior column height of the superior (R1) and inferior (R2) vertebral body was also measured to serve as reference (R = [R1 + R2]/2). Compression rate was given as (1 − A/R) × 100. The wedge angle of fractured vertebral body was defined as the angle between two lines on the superior and inferior endplates of the fractured vertebral body.

At the first visit, written informed consent, demographic information, medical history, medications, smoking status, and vital signs were obtained, and bone density scan, VAS, ODI, EQ-5D questionnaires, and radiologic examinations were conducted. On day 1 (visit 2), vital signs, VAS, ODI, and adverse event monitoring were performed. Visit 3 (at week 1) included vital signs, VAS, ODI, and adverse event monitoring, The fourth and fifth visits, at weeks 4 and 12, involved vital signs, VAS, ODI, EQ-5D, X-ray, and adverse event monitoring. Adverse events (AEs), vital signs, and physical examination were assessed. An adverse event is defined as a compression rate of 80% or more or a VAS pain score of 8 or higher.

### 2.3. Study Treatment

The practitioner of core decompression in this trial was an experienced orthopedic surgeon (S.M.P., with a 10-year experience in orthopedic surgery). Patients in the core decompression group were placed in a prone position. The fractured vertebrae were identified and marked under C-arm guidance. Under local anesthesia, a Jamshidi needle was used to perforate the fractured vertebrae. The needle was carefully inserted through the skin and soft tissues layer-by-layer with continuous monitoring through the pedicle using C-arm guidance. The core wire was extracted from the needle and a syringe was connected to the needle. Subsequently, the plunger of the syringe was gradually withdrawn, resulting in the evacuation of blood and intraosseous decompression. Subsequently, the needle was removed and an aseptic dressing was applied to the puncture site.

In the conservative treatment group, the patients received a regimen that included bed rest, oral pain medication, use of external braces, and ambulation based on their pain tolerance.

In both groups, pain management followed a stepladder approach starting with non-narcotics, low-dose narcotics, and high-dose narcotics. Pain treatment was categorized following the World Health Organization analgesic ladder as 0 (no prescription), 1 (non-narcotic analgesics, acetaminophen, NSAIDs), 2 (low-dose narcotics), and 3 (high-dose narcotics). Esomeprazole, a proton pump inhibitor, was used for medication-induced dyspepsia.

### 2.4. Statistical Analysis

The degree of effectiveness of core decompression performed in this trial has not been previously reported. Therefore, the required sample size was calculated assuming that an effect similar to that of VP performed in general VCFs can be expected. Considering a minimal clinically significant difference of 1.5 on the VAS, a standard deviation of 2.5 in both groups (with an alpha value of 0.05 and a beta value of 0.2), and a tracking loss rate of 0.1, a sample size of 49 individuals per group is required.

For the primary analyses, we used an intention-to-treat strategy, and treatment effects and confidence intervals were calculated using analysis of covariance (ANCOVA) models with adjustment for baseline values of the outcome measure and age. In addition, we used analysis of variance for repeated measures (RM-ANOVA) to compare the ODI score, EQ-5D score, compression rate, and wedge angle. SPSS (version 27.0; IBM Corp., Armonk, NY, USA) was used for all statistical analyses.

## 3. Results

**Figure 1:**
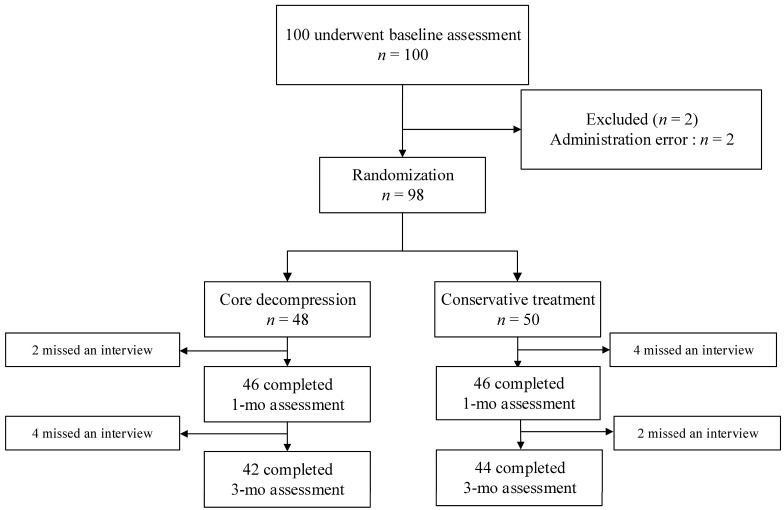
Enrollment and outcomes.

From June 2017 to May 2020, a total of 98 patients were enrolled and underwent randomization. Of the 98 patients, 48 and 50 were assigned to the core decompression and conservative treatment group, respectively. Both groups had six patients each who were lost to follow-up before the 3-month assessment (Figure 1). Demographic and baseline characteristics are shown in Table 1. The core decompression group consisted of 35 females and 13 males, with ages ranging from 57 to 88 years of age (mean age, 76.3 years). The conservative group comprised 38 females and 12 males, with ages ranging from 59 to 91 (mean age, 79.9 years). Most patients had a single fractured vertebral body. The number of patients requiring opioid analgesics for initial pain management was 31 (64.6%) in the core decompression group and 29 (58%) in the conservative group. In Table 1, comorbidities influencing pain perception included diabetes mellitus and polyneuropathy, and medications influencing pain perception included gabapentin and pregabalin.

**Figure 2:**
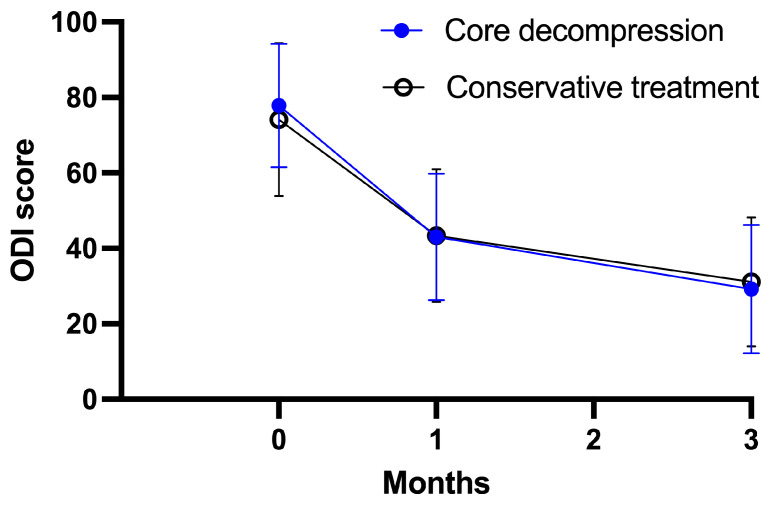
Primary outcome measures through study period.

The study groups did not differ significantly with respect to the primary outcome (VAS score) at 1 month and 3 months. At 1 month, the mean VAS score in the core decompression group was 43.0 ± 16.8, as compared with 43.4 ± 17.6 in the conservative treatment group (adjusted treatment effect: −0.1; 95% confidence interval [CI]: −7.5 to 7.3; *p* = 0.970). At 3 months, the mean VAS score in the core decompression group was 29.2 ± 17.0, as compared with 31.1 ± 17.1 in the conservative treatment group (adjusted treatment effect: 2.0; 95% CI: −5.6 to 9.6; *p* = 0.601) (Table 2, Figure 2). Both groups exhibited substantial improvement in reported back pain after treatment, with similar levels of improvement observed in both groups. There was no significant difference in opioid use rates between the core decompression group and the conservative group before and after treatment in terms of pain medication usage. (Baseline: 64.6% vs. 58.0%, 3-month: 8.3% vs. 8.0%, respectively). In addition, there was a significant reduction observed in opioid use from baseline to 3-month follow-up in both groups. (*p* < 0.001).

**Figure 3:**
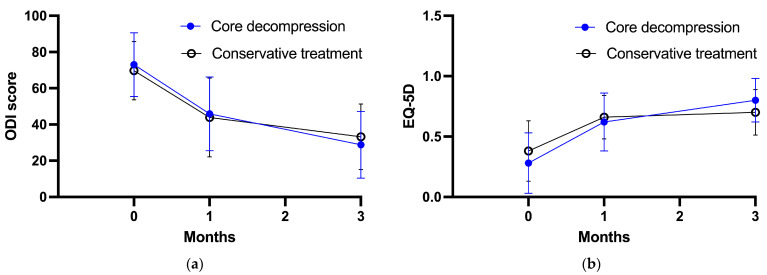
Secondary outcome measures through study period. (**a**) ODI score, (**b**) EQ-5D score.

Figure 3 shows the changes in the secondary outcomes over the course of the follow-up period in both groups. The baseline ODI scores for the core decompression group and the conservative group were 73.0 ± 17.6 and 69.7 ± 16.1, respectively. After 1 month of treatment, the ODI scores were 45.9 ± 20.4 for the core decompression group and 43.9 ± 21.8 for the conservative group. At the 3-month follow-up, the ODI scores showed further improvement, with values of 28.8 ± 18.4 for the core decompression group and 33.2 ± 18.1 for the conservative group. As for the EQ-5D scores, at baseline, the core decompression group scored 0.28 ± 0.25, while the conservative group scored 0.38 ± 0.25. After 1 month of intervention, the EQ-5D scores were 0.62 ± 0.24 for the core decompression group and 0.66 ± 0.18 for the conservative group. At the 3-month follow-up, the EQ-5D scores had improved further, reaching 0.80 ± 0.18 for the core decompression group and 0.70 ± 0.19 for the conservative group. The RM-ANOVA results indicated no significant intergroup differences in the ODI and EQ-5D scores (*p* = 0.917 and *p* = 0.704, respectively). This indicates that the ODI and EQ-5D scores did not differ significantly between the core decompression and conservative treatment groups throughout the follow-up period.

Radiological outcomes are summarized in Table 3. At baseline, compression rates in the core decompression and conservative treatment groups were measured as 28.4 ± 18.4 and 28.8 ± 21.0, respectively. After 3 months, the compression rates for the core decompression and conservative treatment groups were 42.2 ± 22.8 and 36.6 ± 26.0, respectively. Although both treatment groups showed compression progression, there was no significant difference between the groups (*p* = 0.560). Similar results were observed for the wedge angle. At baseline, the wedge angle in the core decompression and conservative treatment groups were 12.2 ± 7.5 and 13.0 ± 7.1, respectively. After 3 months, the wedge angles in the core decompression and conservative treatment groups were 15.8 ± 9.0 and 14.0 ± 7.6, respectively. There were no significant differences between the study groups (*p* = 0.753).

No serious adverse events, resulting in life-threatening conditions or severe sequelae, were observed.

## 4. Discussion

This randomized controlled trial aimed to compare the effectiveness and safety of a newly designed vertebral body core decompression technique with those of conventional conservative treatment in patients with painful VCFs. The results indicated that the core decompression group did not demonstrate a significant difference in pain improvement compared with the conservative treatment group. Moreover, there were no significant differences in the measures of disability and quality of life between the two groups. Additionally, no significant differences were observed in the radiological parameters of the study groups at baseline and 3 months.

The core decompression technique employed in this study was based on the principles of the widely used core decompression technique for ONFH [16]. Previous studies have shown that core decompression in ONFH can reduce intraosseous pressure and improve blood flow, facilitating bone remodeling and pain reduction [17,18,19]. Several studies have demonstrated the association between fracture pain, and increased intraosseous pressure has been demonstrated in various studies [20,21,22,23]. In addition, several studies found significantly higher intraosseous pressure in patients with VCFs than in those with normal vertebral bodies [10,20]. Thus, this study aimed to investigate whether decompression of the vertebral body can alleviate VCF-associated pain. The findings indicated a decrease in pain VAS scores in the core decompression group up to 3 months after treatment, but no significant difference in the treatment effect was found compared to that of conservative treatment. Furthermore, several studies have shown that VCFs have a negative impact on quality of life, even after the fracture has healed, resulting in worse disability and quality of life outcomes [24,25]. In this study, the analysis of quality of life and disability recovery in the core decompression group did not reveal a significant difference compared to the conservative treatment group.

A few studies have conducted procedures similar to the core decompression performed in this study, referred to as vertebral body perforation, and have reported favorable results. Ogihara et al. demonstrated that vertebral body perforation provides sufficient pain relief in patients with painful lumbar compression fractures [26]. They attributed pain relief to improved blood flow, equalization of intraosseous pressure, reduced stimulation of pain nerve fibers, and decreased levels of pain-related substances. Additionally, a comparative study of VP and vertebral body perforation conducted by Yokoyama et al. suggested that vertebral body perforation could be a viable alternative to VP in patients with a vertebral body height loss of 30% or less, as it demonstrated an efficacy similar to VP without concerns associated with cement usage [27]. However, given the lack of advantages of core decompression over conservative treatment observed in this study, the suitability of core decompression as an alternative to VP is questionable. Considering the similarities in the process and invasiveness of core decompression and VP, VP may be preferable based on several studies that have demonstrated improved outcomes compared to conservative treatment [2,28].

This study has several limitations. First, we were unable to compare the intraosseous pressure of patients in the conservative treatment and core decompression groups, despite recognizing the importance of intraosseous pressure in the pain reduction effect of core decompression. Second, patients’ knowledge of their treatment assignments may have influenced their responses to the questionnaire. Third, the follow-up period was limited to 3 months. Given that pain and disability resulting from vertebral compression fractures can affect a patient’s quality of life over an extended period, a short follow-up duration may not fully capture the long-term effects.

## 5. Conclusions

In this prospective randomized controlled trial involving patients with osteoporotic VCF, we found no beneficial effects in performing core decompression of the vertebral body compared with that of conservative treatment in patients with painful VCFs.

## Figures and Tables

**Table 1:** Demographics and baseline characteristics.

	Core Decompression Group *n* = 48	Conservative Group *n* = 50
Age (year)	76.3 ± 6.7	79.9 ± 6.6
Female sex—No.	35 (72.9%)	38 (76%)
Height (cm)	156.0 ± 7.0	154.6 ± 8.2
Weight (kg)	60.8 ± 11.2	54.9 ± 8.5
BMI ^a^	24.9 ± 4.0	23.0 ± 3.0
Vitamin D (ng/mL)	21.43 ± 12.30	22.40 ± 10.96
No. of fractured vertebral bodies—mean	1.02	1.06
One—no.	47	47
Two—no.	1	3
Vertebral level		
T10–T12	15 (30.0%)	18 (34.0%)
L1–L2	24 (48%)	28 (52.8%)
L3–L5	11 (22.0%)	7 (13.2%)
Initial pain management		
0–1 (no prescription~non-opioid analgesics)	17	21
2–3 (opioid analgesics)	31	29
Comorbidities influencing pain perception	4 (8.3%)	4 (8.0%)
Concomitant medication		
Steroid usage	0	0
Medications influencing pain perception	3 (6.2%)	4 (8.0%)
VAS ^b^ score	77.9 ± 16.4	74.1 ± 20.3
ODI ^c^ score	72.8 ± 17.8	70.1 ± 15.9
EQ-5D ^d^ score	0.30 ± 0.25	0.37 ± 0.26

^a^ body mass index; ^b^ visual analogue score; ^c^ Oswestry disability index; ^d^ European quality of life-5 dimension.

**Table 2:** Primary outcome measures.

	Core Decompression Group	Conservative Treatment Group	Treatment Effect (95% CI) *	*p*-Value
VAS				
At baseline	77.9 ± 16.4	74.1 ± 20.3		
At 1 month	43.0 ± 16.8	43.4 ± 17.6	−0.1 (−7.5 to 7.3)	0.970
At 3 months	29.2 ± 17.0	31.1 ± 17.1	2.0 (−5.6 to 9.6)	0.601

* Between-group comparisons, confidence intervals, and *p*-values were calculated with the use of analysis-of-covariance models with adjustment for baseline value of the outcome measure and age. Negative treatment effects favor core decompression, and positive treatment effects favor the control.

**Table 3:** Radiographic outcome measures.

	Core Decompression Group	Conservative Group	*p*-Value
Compression rate (%)			0.560
At baseline	28.4 ± 18.4	28.8 ± 21.0	
At 3 months	42.2 ± 22.8	33.6 ± 26.0	
Wedge angle (degrees)			0.753
At baseline	12.2 ± 7.5	13.0 ± 7.1	
At 3 months	15.8 ± 9.0	14.0 ± 7.6	

Between-group comparisons, confidence intervals, and *p*-values were calculated with the use of RM-ANOVA.

## Data Availability

The data can be obtained from the corresponding author upon reasonable request.

## References

[1] Cohen L.D. (1990). Fractures of the osteoporotic spine. Orthop. Clin. N. Am..

[2] Farrokhi M.R., Alibai E., Maghami Z. (2011). Randomized controlled trial of percutaneous vertebroplasty versus optimal medical management for the relief of pain and disability in acute osteoporotic vertebral compression fractures. J. Neurosurg. Spine.

[3] Jang H.D., Kim E.H., Lee J.C., Choi S.W., Kim H.S., Cha J.S., Shin B.J. (2022). Management of Osteoporotic Vertebral Fracture: Review Update 2022. Asian Spine J..

[4] Cho M.J., Moon S.H., Lee J.H., Lee J.H. (2021). Association between Osteoporotic Vertebral Compression Fractures and Age, Bone Mineral Density, and European Quality of Life-5 Dimensions in Korean Postmenopausal Women: A Nationwide Cross-sectional Observational Study. Clin. Orthop. Surg..

[5] Alexandru D., So W. (2012). Evaluation and management of vertebral compression fractures. Perm. J..

[6] Prather H., Hunt D., Watson J.O., Gilula L.A. (2007). Conservative care for patients with osteoporotic vertebral compression fractures. Phys. Med. Rehabil. Clin. N. Am..

[7] Cho S.T., Kim S.J., Nam B.J., Kim K.W., Lee G.H., Kim J.H. (2022). Absolute Bed Rest Duration of 3 Days for Osteoporotic Vertebral Fractures: A Retrospective Study. Asian Spine J..

[8] Park S.M., Park C., Kim H., Kim H.J., Yeom J.S., Lee C.K., Chang B.S. (2018). Is Redo Vertebroplasty an Effective Treatment on the Same Vertebra?. CardioVascular Interv. Radiol..

[9] Park S.M., Park J.W., Kim H., Kim H.J., Yeom J.S., Lee C.K., Chang B.S. (2018). Morphological changes of vertebral compression fracture with intra-vertebral cleft treated with percutaneous vertebroplasty. J. Orthop. Sci..

[10] Arnoldi C.C. (1976). Intraosseous hypertension. A possible cause of low back pain?. Clin. Orthop. Relat. Res..

[11] Lemperg R.K., Arnoldi C.C. (1978). The significance of intraosseous pressure in normal and diseased states with special reference to the intraosseous engorgement-pain syndrome. Clin. Orthop. Relat. Res..

[12] Yeh M.L., Heggeness M.H., Chen H.H., Jassawalla J., Luo Z.P. (2006). Compressive loading at the end plate directly regulates flow and deformation of the basivertebral vein: An analytical study. J. Orthop. Surg. Res..

[13] Choi J.Y., Park S.M., Kim H.J., Yeom J.S. (2022). Recent Updates on Minimally Invasive Spine Surgery: Techniques, Technologies, and Indications. Asian Spine J..

[14] Pierce T.P., Jauregui J.J., Elmallah R.K., Lavernia C.J., Mont M.A., Nace J. (2015). A current review of core decompression in the treatment of osteonecrosis of the femoral head. Curr. Rev. Musculoskelet. Med..

[15] Kim G.U., Park W.T., Chang M.C., Lee G.W. (2022). Diagnostic Technology for Spine Pathology. Asian Spine J..

[16] Hua K.C., Yang X.G., Feng J.T., Wang F., Yang L., Zhang H., Hu Y.C. (2019). The efficacy and safety of core decompression for the treatment of femoral head necrosis: A systematic review and meta-analysis. J. Orthop. Surg. Res..

[17] Mont M.A., Jones L.C., Hungerford D.S. (2006). Nontraumatic osteonecrosis of the femoral head: Ten years later. J. Bone Joint Surg. Am..

[18] Mont M.A., Ragland P.S., Etienne G. (2004). Core decompression of the femoral head for osteonecrosis using percutaneous multiple small-diameter drilling. Clin. Orthop. Relat. Res..

[19] Zhang H.J., Liu Y.W., Du Z.Q., Guo H., Fan K.J., Liang G.H., Liu X.C. (2013). Therapeutic effect of minimally invasive decompression combined with impaction bone grafting on osteonecrosis of the femoral head. Eur. J. Orthop. Surg. Traumatol..

[20] Arnoldi C.C. (1972). Intravertebral pressures in patients with lumbar pain. A preliminary communication. Acta Orthop. Scand..

[21] Esses S.I., Moro J.K. (1992). Intraosseous vertebral body pressures. Spine.

[22] Ochia R.S., Ching R.P. (2002). Internal pressure measurements during burst fracture formation in human lumbar vertebrae. Spine.

[23] Arnoldi C.C., Linderholm H., Mussbichler H. (1972). Venous engorgement and intraosseous hypertension in osteoarthritis of the hip. J. Bone Joint Surg. Br..

[24] Lyles K.W., Gold D.T., Shipp K.M., Pieper C.F., Martinez S., Mulhausen P.L. (1993). Association of osteoporotic vertebral compression fractures with impaired functional status. Am. J. Med..

[25] Kendler D.L., Bauer D.C., Davison K.S., Dian L., Hanley D.A., Harris S.T., McClung M., Miller P., Schousboe J., Yuen C. (2016). Vertebral Fractures: Clinical Importance and Management. Am. J. Med..

[26] Ogihara M. (2006). Core decompression of vertebral body for osteoporotic vertebral compression fracture. Pain Clinic.

[27] Yokoyama K., Kawanishi M., Yamada M., Tanaka H., Ito Y., Hirano M., Kuroiwa T. (2012). Comparative study of percutaneous vertebral body perforation and vertebroplasty for the treatment of painful vertebral compression fractures. AJNR Am. J. Neuroradiol..

[28] Klazen C.A., Lohle P.N., de Vries J., Jansen F.H., Tielbeek A.V., Blonk M.C., Venmans A., van Rooij W.J.J., Schoemaker M.C., Juttmann J.R. (2010). Vertebroplasty versus conservative treatment in acute osteoporotic vertebral compression fractures (Vertos II): An open-label randomised trial. Lancet.

